# Conventional MRI-Derived Biomarkers of Adult-Type Diffuse Glioma Molecular Subtypes: A Comprehensive Review

**DOI:** 10.3390/biomedicines10102490

**Published:** 2022-10-05

**Authors:** Paola Feraco, Rossana Franciosi, Lorena Picori, Federica Scalorbi, Cesare Gagliardo

**Affiliations:** 1 Neuroradiology Unit, Ospedale S. Chiara, Azienda Provinciale per i Servizi Sanitari, Largo Medaglie d’oro 9, 38122 Trento, Italy; 2 Department of Experimental, Diagnostic and Specialty Medicine (DIMES), University of Bologna, Via S. Giacomo 14, 40138 Bologna, Italy; 3 Radiology Unit, Santa Maria del Carmine Hospital, 38068 Rovereto, Italy; 4 Nuclear Medicine Unit, Ospedale S. Chiara, Azienda Provinciale per i Servizi Sanitari, Largo Medaglie d’oro 9, 38122 Trento, Italy; 5 Nuclear Medicine Unit, Foundation IRCSS, Istituto Nazionale dei Tumori, 20121 Milan, Italy; 6 Department of Biomedicine, Neuroscience and Advanced Diagnostics (BIND), University of Palermo, Via del Vespro 129, 90127 Palermo, Italy

**Keywords:** isocitrate dehydrogenase (IDH), adult diffuse gliomas, magnetic resonance imaging (MRI)

## Abstract

The introduction of molecular criteria into the classification of diffuse gliomas has added interesting practical implications to glioma management. This has created a new clinical need for correlating imaging characteristics with glioma genotypes, also known as radiogenomics or imaging genomics. Although many studies have primarily focused on the use of advanced magnetic resonance imaging (MRI) techniques for radiogenomics purposes, conventional MRI sequences remain the reference point in the study and characterization of brain tumors. A summary of the conventional imaging features of glioma molecular subtypes should be useful as a tool for daily diagnostic brain tumor management. Hence, this article aims to summarize the conventional MRI features of glioma molecular subtypes in light of the recent literature.

## 1. Introduction

The introduction of molecular criteria into the classification of diffuse gliomas has given rise to interesting, wide-ranging implications regarding glioma management. In the current classification, all diffuse gliomas (of astrocytic and non-astrocytic origin) have been grouped based on their growth pattern, clinical behavior, and specifically sharing of the mutational state of the gene that codes for isocitrate dehydrogenase (IDH) in its isoforms (IDH1 and IDH2) [1,2].

Mutant IDH genes produce proteins that convert α-ketoglutarate to the putative oncometabolite 2-hydroxyglutarate (2HG) [3]. IDH mutations have been demonstrated to be one of the earliest events in glioma formation. The resulting production of 2HG appears to drive extensive epigenetic changes, thereby altering cellular differentiation and contributing to oncogenesis. These molecular parameters currently constitute a crucial component of glioma diagnosis, providing a combined phenotypic and genotypic diagnosis. Thus, a new clinical need has arisen to correlate imaging characteristics with glioma genotypes, also known as *radiogenomics* or *imaging genomics*.

Regardless of grade, the first phase in glioma molecular characterization is IDH testing [4]. Mutations in IDH1 and IDH2 are associated with a significant increase in progression-free survival (PFS) and overall survival (OS) [5,6]. They are present in 50–80% of grade 2 and 3 astrocytic and oligodendroglial tumors. In the current classification, however, all IDH-mutant (IDH-MUT) diffuse astrocytic tumors are considered a single type (Astrocytoma, IDH-mutant) and are then graded as WHO grade 2, 3, or 4 [2]. An immunohistochemical investigation of the most frequent IDH1 mutation (R132H) should be routinely performed on all tissue samples where diffuse glioma is suspected. This is primarily because the test can be decisive in the differential diagnosis between infiltrating astrocytoma and reactive gliosis [4,7].

Since 2016 [1], the diagnosis of oligodendroglioma and anaplastic oligodendroglioma has required the concomitant deletion of the short arm of chromosome 1 and the long arm of chromosome 19 (1p/19q codeletion); this is in addition to the demonstration of an IDH mutation. This genotypic feature is due to an unbalanced translocation between chromosomes 1 and 19 and it is a powerful predictor of both the response to therapy and survival [8]. Therefore, its presence must be ascertained in all tumors with oligodendroglial differentiation. On the contrary, all diffuse astrocytic gliomas without an IDH mutation are known as IDH-wildtype (IDH-WT), and they can be considered glioblastomas (GBMs) [2,9,10].

IDH-MUT GBMs are now considered astrocytoma IDH-MUT grade 4 [2]; they are uncommon and assumed to arise from low-grade gliomas and have improved outcomes compared to IDH-WT GBMs [11].

Moreover, in the 2016 WHO classification, H3G34-mutant diffuse gliomas were not considered a distinct tumor type, and most cases are currently classified as IDH-WT GBMs [1]. However, despite it having recently been recommended to consider H3G34-mutant diffuse gliomas as a new WHO grade 4 pediatric tumor type, which should be referred to as “diffuse hemispheric glioma, H3G34-mutant” [2,12], this kind of tumor can also affect young adults and should be recognized.

In addition, O6-methylguanine-DNA-methyltransferase (MGMT) promoter methylation status is of great clinical importance since it has been demonstrated to be associated with improved outcomes in patients with GBM. It may also be a predictive marker of sensitivity to chemotherapy [13,14].

The integrated use of phenotypic and genotypic parameters permits the definition of more homogeneous diagnostic categories with greater objectivity than in the past. Thus, it will consequently be possible to establish more precise correlations between the prognosis and response to treatment of such neoplasms. Specifically, four prognostic groups for imaging research pertaining to gliomas can be recognized: IDH status, 1p/19q status, MGMT status, and H3G34 status.

Although many recent studies have focused on using advanced MRI techniques (perfusion, spectroscopy, and machine learning techniques) for radiogenomic purposes [15,16,17], conventional MRI sequences remain the reference point in the study and characterization of brain tumors. Moreover, since it remains a challenge to standardize post-processing methods regarding advanced techniques and computational imaging approaches (such as machine learning), the utility of a review of conventional imaging features of glioma molecular subtypes should be helpful as a tool for daily diagnostic brain tumor management. Although most of the literature on this topic was published in the last five years, it is continuously growing, and to date, a restricted number of reviews have been published. Hence, this article aimed to summarize the conventional imaging features relating to glioma molecular subtypes in light of the recent literature. Sequences were considered “conventional”, in line with recently published guidelines regarding glioma imaging [18], as applied to the study of diffuse brain gliomas. 

## 2. Materials and Methods

Extensive research of the English-language literature was performed in April 2022 using the PubMed database (https://pubmed.ncbi.nlm.nih.gov, accessed on 10 April 2022), and employing the following keywords and their combinations: “diffuse glioma”, “glioblastoma”, “astrocytoma”, “oligodendroglioma”, AND “isocitrate dehydrogenase”, “IDH”, “1p19q”, “H3G34”, “MGMT” AND “magnetic resonance imaging”, “MRI.” Preclinical and clinical studies from the last five years (January 2016–April 2022) were carefully reviewed, focusing on the different diffuse glioma molecular subtypes. The publication date was restricted to the last six years since molecular criteria were introduced into the classification of diffuse gliomas in 2016 [1]. Various articles within this time range were included to maximize this review’s topic coverage. Full texts in English were included, together with the most significant corresponding references. The exclusion criteria were the unavailability of the full text; non-English publication; case report; letter; commentary; review; editorial; no interpretation of conventional MRI (cMRI) sequences; studies limited to advanced MR sequences or machine learning; and studies devoid of glioma molecular subtype information. After this, the results were assessed according to the PRISMA statement [19]. Conventional MRI findings were described from the included studies, after which they were systematically organized and grouped according to the particular field of study and perspective. 

## 3. Results

One hundred and six articles were identified from the PubMed literature search. These were subsequently screened for relevance: 66 studies were excluded according to the exclusion criteria, whereas 45 were included. The full texts were available for all of the 45 included studies, which were also included in the qualitative analysis. Considering the different molecular subgroups, the following were identified: 27 articles relating to cMRI in IDH and 1p/19q codeletion expression, 12 regarding cMRI in MGMT promoter methylation status, and 6 discussing cMRI findings in H3G34-mutated tumors.

## 4. Discussion

The MR findings regarding the molecular subgroups (IDH, 1p/19q-codeletion, MGMT, and H3G34) were grouped according to the principal reported descriptive characteristics: location, borders, internal signal characteristics, contrast enhancement, T2-weighted (T2w)/fluid-attenuated inversion recovery (FLAIR) mismatch sign, and diffusion-weighted imaging (DWI).

The principal morphological MRI features associated with each of the different prognostic groups (IDH, 1p/19q-codeletion, MGMT, and H3G34) highlighted in this review have been grouped and are shown in Table 1.

### 4.1. Conventional MRI and DWI Findings

#### 4.1.1. Location

**Figure 1:**
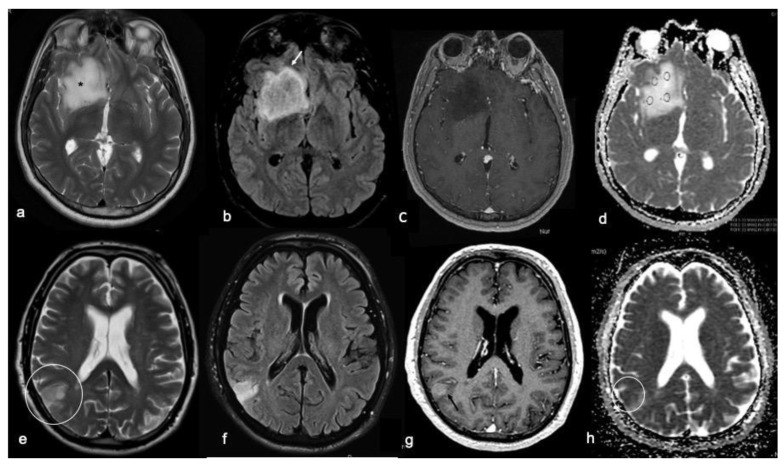
Radiological characteristics of IDH-MUT (top row: (**a**–**d**)) and IDH-WT (bottom row: (**e**–**h**)) diffuse gliomas. For each case, T2w fast spin echo (**a**,**e**), FLAIR sequences (**b**,**f**), T1w fast spin echo contrast-enhanced (**c**,**g**), and ADC map (**d**,**h**) images are shown. (**a**–**d**) Right frontal tumor with defined borders, a high and homogeneous T2w signal (asterisk in (**a**)), a T2w/FLAIR mismatch sign (arrow in (**b**)), no contrast enhancement, and high ADC values; these are the typical imaging findings of an IDH-MUT glioma. (**e**–**h**) Right parietal ill-defined tumor (inside the circle in (**e**,**h**)) characterized by a low T2w signal (**e**), no evidence of a T2w/FLAIR mismatch sign (**f**), a slight contrast enhancement (**g**), and a low signal in the ADC maps; these imaging findings are typical of an IDH-WT glioma.

Diffuse gliomas can be found in different locations, which reflects their tumor cell origin. Different molecular subgroups have been demonstrated with a distinctive anatomic distribution, which is of great importance in patient diagnosis and clinical outcomes. This kind of anatomic feature is consistent with its corresponding histological subtypes. Considering the IDH mutational status, IDH-MUT tumors have been widely reported as being more frequently located in the frontal region than the temporo-insular region [20,21] and also in the bilateral insular lobes [22]. Moreover, subventricular zone involvement has also been related to IDH-MUT tumors [23]. Tumors with the coexistence of IDH1 and telomerase reverse transcriptase (TERT) gene promoter mutations are inclined to grow in the left frontal lobe and close to the midline region. On the contrary, a TERT-only mutated glioma is more deeply located than IDH-MUT and 1p/19q codeleted tumors [22]. However, a non-lobar location and multifocal/multicentric distribution have been described as independent predictors of IDH-WT gliomas [24], although they have been described in the basal ganglia and rostral areas of the hemispheres and are usually closer to midline structures [25] (Figure 1). 

Considering tumors with IDH-MUT and 1p/19q codeletion, most are located in the frontal lobes and cortex [25] and the anterior extensions of the lateral ventricles compared to IDH-WT astrocytomas. 

**Figure 2:**
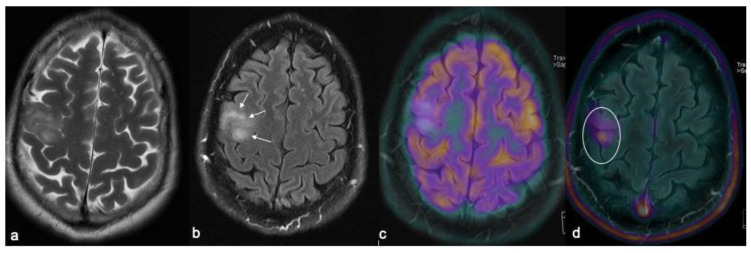
IDH-WT glioma with ill-defined borders in the posterior aspect of the middle frontal gyrus showing an inhomogeneous high signal on the T2w fast spin echo (**a**) and FLAIR (arrows in (**b**)) sequences with no radiotracer uptake at FDG-PET (**c**), but demonstrating a noticeable uptake upon FET-PET examination (circle in (**d**)).

Referring to MGMT promoter methylation, methylated GBMs have been reported to be hemispheric, with a preference for being lateralized on the left side, whereas unmethylated GBMs are lateralized in the right hemisphere (Figure 2) [26,27].

Also known as diffuse hemispheric gliomas, H3G34 MUT tumors are usually supratentorial with predominant involvement of the fronto-parietal lobes [12,28]. These particular tumors are all voluminous (major axis > 3 cm at onset) and have a considerable mass effect. The corpus callosum and basal ganglia are involved in one-third of cases. Leptomeningeal spread has been frequently observed, which is often associated with ependymal contact [28]. The involvement of up to three cerebral lobes has been also reported in cases of the most voluminous tumors, as well as synchronous tumors in the cerebral hemisphere and posterior fossa [29,30]. A multi-lobular extension, which is similar to the pattern of gliomatosis cerebri, has also been observed, whereas midline involvement has been observed as an extension of a primarily hemispheric tumor [30].

#### 4.1.2. Borders

Tumor borders are an important characteristic regarding glioma management since their appearance is critical in defining surgery management. Lesions with sharp borders have been frequently related to IDH-MUT, whereas tumors with indistinct borders are more frequently IDH-WT and 1p/19q non-codeleted. In detail, an evaluation of the borders on FLAIR images has enabled the discrimination between IDH-WT and IDH-MUT tumors, thereby demonstrating the ill-defined borders of IDH-WT [20] (Figure 1 and Figure 2).

Moreover, poorly defined, non-enhancing margins are an independent predictor of IDH1-WT [24]. Considering MGMT promoter methylation, indistinctly enhancing tumor margins have been observed more frequently in methylated than unmethylated gliomas (45.5% versus 7.7%, respectively) [31]. 

There is some disagreement in the literature regarding the border description of H3G34 tumors; indeed, these tumors have been described as well-delineated with a peripheral T2 hypersignal [28], as well as poorly delimited with infiltrative lesions [12]. In some cases, patients with a misleading radiological presentation typically present with an MRI aspect that was previously described as “gliomatosis cerebri-like.” This consists of a non-contrast, enhancing, ill-defined, infiltrating lesion that led the physician to suspect an alternative diagnosis to a high-grade glioma due to the young age of the patient [12,28]. However, more studies with larger patient samples are required in order to improve the characterization of this molecular category.

#### 4.1.3. Internal Signal Characteristics 

**Figure 3:**
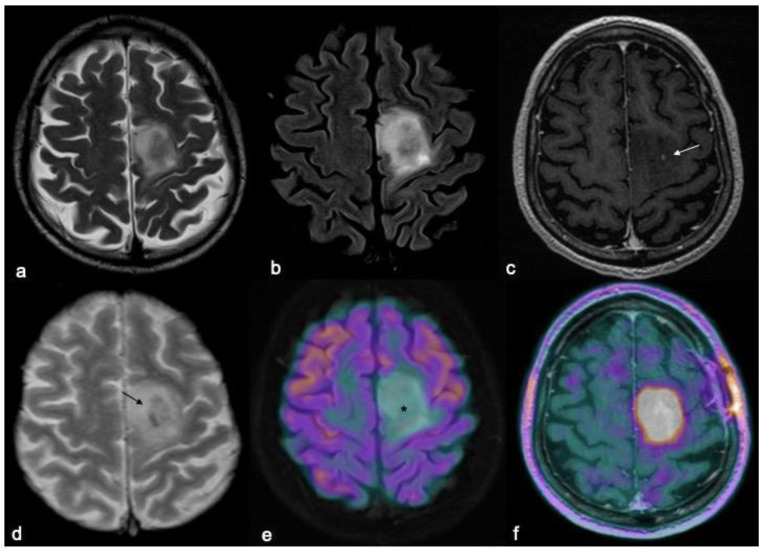
MRI and PET characteristics of a diffuse IDH-MUT 1p/19q codeleted glioma (oligodendroglioma grade 2). T2w fast spin echo (**a**), T2w FLAIR (**b**), T1w fast spin echo contrast-enhanced (**c**), T2*-weighted gradient echo (**d**), FDG-PET/MRI (**e**), and DOPA-PET/MRI (**f**) images are shown. Mesial frontal mass with a low mass effect and without any significant enhancements after contrast (arrow in (**c**) indicates the only spot of blood–brain barrier leakage), and some intratumoral calcifications (arrow in (**d**); CT not shown) with no significant radiotracer uptake by FDG-PET (asterisk in (**e**)), but showing a lively uptake of the PET-DOPA radiotracer (**f**).

Considering the internal consistency of the tumors, IDH-WT tumors are more necrotic than IDH1-MUT ones [32,33] and might present hemorrhage [33,34]. Assessment of the tumors on T2w images may assist in making a differential diagnosis. Indeed, a homogeneous T2w signal has been demonstrated to be related to IDH-MUT 1p/19q non-codeleted tumors [24]. The evaluation of T2w images, particularly the use of a quantitative approach through T2 mapping sequences, has recently shown how the T2 signal is significantly increased in IDH-MUT gliomas compared to the wild-type; this may be due to an accumulation of 2HG and modified tumor metabolism [35]. On the contrary, calcifications within IDH-MUT are more likely related to IDH-MUT 1p/19q codeleted tumors [24], which bear a heterogeneous signal intensity in T2w images with a significantly higher micro-vascularity and higher vascular heterogeneity (Figure 3) [36].

Such tumors also show more pial invasion compared to the IDH1-MUT non-codeleted group [24].

Of interest, the combined evaluation of the T2w and FLAIR images (also known as “T2/FLAIR mismatch sign”) has recently emerged as a non-invasive biomarker of IDH-MUT 1p/19q non-codeleted gliomas (Figure 1). This sign is characterized by suppression on the FLAIR images of the high T2w lesion signal with the exclusion of the borders. The applicability of this sign seems to be limited to lower-grade gliomas, albeit constituting a highly specific imaging biomarker for the IDH-MUT 1p/19q non-codeleted molecular subtype [22,37,38,39,40]. Moreover, the combination of the T2/FLAIR mismatch sign and the apparent diffusion coefficient (ADC) parameters can improve the identification of IDH-MUT 1p/19q non-codeleted diffuse lower-grade gliomas [40]. 

**Figure 4:**
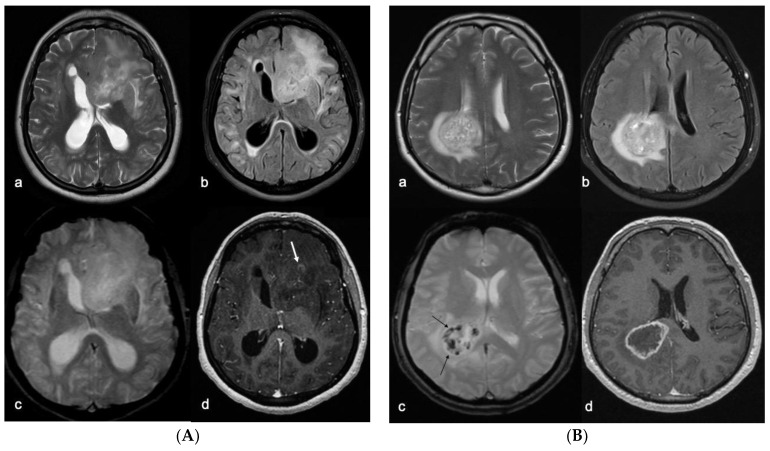
(**A**) Left frontal GBM with typical imaging findings of MGMT methylated (inactive) tumors. Despite the size of the lesion and its heterogeneous signal on the T2w fast spin echo (**a**) and T2w FLAIR images (**b**), there are no calcifications or intratumoral bleedings on the T2*w gradient echo images (**c**) and only a small irregular area of contrast enhancement appreciable after i.v. administration of contrast medium on the T1w pulse sequences (arrow in (**d**)). (**B**) Right GBM with typical imaging findings of the MGMT not-methylated subtype. The lesion is deeply localized in the fronto-parietal periventricular zone, with a heterogeneous signal on the T2w fast spin echo (**a**) and T2w FLAIR images (**b**), calcifications and/or bleedings on the T2*w gradient echo images (arrows in (**c**)), with a typical ring enhancement after i.v. administration of contrast medium on the T1w pulse sequences (**d**) that delimits a central area of intratumoral necrosis.

Similarly, an evaluation of cerebral microbleeds in gliomas has proved to be of significant value in predicting the glioma grade, IDH1 mutation, and MGMT methylation; this is affected by evaluating intratumoral susceptibility signal (ITSS) grades. Indeed, IDH1-MUT and MGMT methylated tumors have significantly lower ITSS grades than IDH-WT and unmethylated MGMT gliomas (Figure 4). 

No relationship between 1p/19q deletion status and ITSS grades has yet been detected [41]. 

Gliomas with methylated MGMT promoters display less edema than MGMT promoter unmethylated glioblastoma [25,42,43]. In contrast, a greater degree of edema/necrosis and tumor/necrosis volume ratios has been observed in unmethylated MGMT, in agreement with the aggressive nature of these tumors [44]. Furthermore, it has also been reported that a higher rate of MGMT promoter protein expression is related to less necrosis in GBM compared to those cases negative for or with a lower rate of MGMT promoter protein expression [27]. 

**Figure 5:**
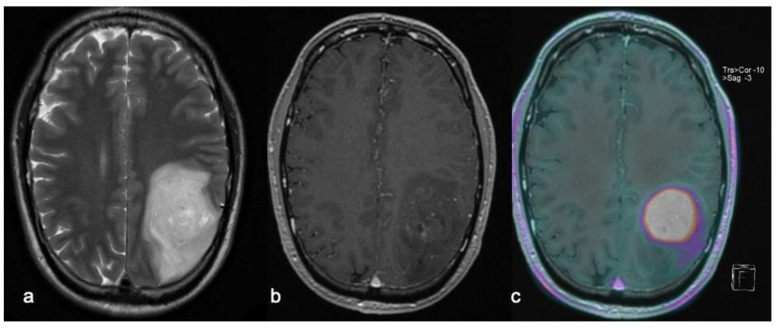
Left parietal H3G34 glioma with little perilesional edema appearance on T2w fast spin echo imaging (**a**) and focal slight contrast enhancement after Gd-based contrast media injection on T1w sequences (**b**), showing vivid radiotracer uptake upon FET-PET imaging (**c**).

The MRI features of diffuse gliomas with an H3G34 mutation may present with a marked degree of heterogeneity in some cases while not even fulfilling the imaging criteria of high-grade gliomas [45]. These tumors generally present as being cortico-subcortical (81%), poorly delimited (69%), and with infiltrative lesions [11]. An intratumoral, pre-contrast, T1-weighted hypersignal is frequently observed in diffuse gliomas with an H3G34 mutation [28]. Indeed, these tumors typically hemorrhage at onset [12,45]. Calcifications might be observed in some patients, as reported by the two largest published retrospective studies [28,30]. Although a mass effect and cystic components were identified in half of the cases, no peritumoral edema has been described in relation to the lesions [28]; edema has seldom been reported in H3G34 tumors (Figure 5). 

#### 4.1.4. Contrast Enhancement

Contrast enhancement (CE) is another important feature in exploring the possible diagnosis of a brain tumor and has been demonstrated as being associated with OS [46]. Indeed, no enhancements and a smooth, non-enhancing margin, as revealed by MRI, are predictive of a longer PFS and OS in lower-grade tumors [47,48]. On the contrary, a larger proportion of enhancing tumors has been shown to be more frequently related to IDH-WT [24], thereby indicating an unfavorable prognostic factor for this group [45]. CE has been shown to be more frequent in IDH-MUT and 1p/19q codeleted tumors [49] than in non-codeleted IDH-MUT tumors [50]. Indeed, after intravenous contrast administration, 1p/19q codeleted tumors generally do not enhance, although a patchy, multifocal enhancement with a dot-like or lacy pattern has been reported in up to 50% of cases (Figure 3) [36]. This might assist in differentiating low-grade non-codeleted IDH-MUT from 1p/19q codeleted IDH-MUT tumors.

Mixed-nodular CE in high-grade tumors (GBMs) has been demonstrated to be more frequent in MGMT promoter methylated glioblastoma, whereas ring enhancement is most common in MGMT promoter unmethylated glioblastomas (Figure 4) [26,51]. A recent study did not establish a significant association between the existence of enhancing tumor margins and the status of MGMT promoter methylation [52]. H3G34 tumors generally demonstrate the absence of or only a faint contrast enhancement (Figure 5) [30,53].

#### 4.1.5. DWI

Conventional MRI is invaluable for glioma genotyping, particularly regarding tumors that are presumed to be lower grade. In addition to basic MRI protocols, DWI is routinely performed in MRI glioma protocols [18] and it might be considered a conventional sequence, which is worthy of a separate discussion. In the last five years, much research has been conducted in order to evaluate the role of DWI and ADC maps, in isolation or in combination with other techniques, in the routine evaluation of gliomas. The addition of quantitative measures of ADC values to conventional MR imaging could be used as a non-invasive marker of specific molecular patterns since IDH-WT and IDH-MUT gliomas display significantly different ADC values (Figure 1) [49,54]. Indeed, ADC values might distinguish between intact IDH-MUT group gliomas, which show significantly higher values than IDH-MUT 1p/19q codeleted and IDH-WT tumors [55]. IDH1-mutant 1p/19q-codeleted tumors frequently have mixed/restricted diffusion characteristics, thereby proving difficult to separate from IDH-WT [24].

Referring to MGMT, ADC values have been shown to be higher in methylated GBMs than in unmethylated ones, with an ADC ratio that is significantly higher in methylated versus unmethylated tumors [31,56]. Higher ADC values may predict MGMT promoter methylation status [57] and a longer overall survival rate [50], thereby reflecting lower tumor cellularity in the methylated group. The majority of H3G34-mutated tumors show areas of ADC restriction on DWI at onset [12,28]. 

## 5. Conclusions

The associations between the various tumor molecular subtypes and cMRI imaging findings provide a new opportunity for the non-invasive prediction of molecular genetics in gliomas. 

The use of advanced MRI techniques enables key insights beyond the gross anatomy of tumors, providing reliable information on the involved white matter pathways via the use of tractography reconstructions from diffusion tensor imaging (DTI), brain eloquent area functionality based on blood oxygen level-dependent (BOLD) imaging (i.e., functional MRI (fMRI)), tissue metabolism by hemodynamic parameters analyzed with perfusion-weighted imaging (PWI) techniques, and microstructural tissue characteristics such as stiffness/softness via the use of MR-elastography of tumor features invisible to even the most experienced radiologist and evaluated exclusively through radiomics analyses techniques. Although there is increasing use of and interest in these techniques, they still lack wide standardization across software, vendors, and institutions; furthermore, they are not easily accessible outside the academic world since they may require additional software, workstations, or dedicated devices. In this continuously expanding field, cMRI still represents a mandatory and extremely important step in the diagnostic workflow of neuro-oncological patients for preoperative diagnosis, treatment planning, and subsequent follow-up. Even if the value of cMRI should not be underestimated, the integration of advanced MRI techniques should always be considered where available. Nevertheless, the association between cMRI and the molecular subtype of gliomas is nowadays already proving to form an important basis for preoperative, personalized, surgical treatment, which is based on molecular pathology.

## Figures and Tables

**Table 1:** The principal morphological MRI features associated with IDH groups, 1p/19q-codeletion, MGMT, and H3G34. SI, signal intensity; Meth, methylated; UnMeth, unmethylated; ADC, apparent diffusion coefficient; ITSS, intratumoral susceptibility signal.

Type	Location	Borders	Internal Signal Characteristics	Contrast Enhancement	DWI
IDH-Mut	Frontal > temporal	Sharp	Homogenous with high SI on T2w; T2/Flair mismatch sign; low ITSS grade	Infrequent	>ADC values than IDH-WT and 1p/19q-codeleted
IDH-WT	No prevalence; close to midline	Indistinct	Necrosis and hemorrhage; high ITSS grade	More frequent than IDH-Mut	<ADC values than IDH-Mut
IDH-Mut and 1p/19q-codeletion	Frontal	Indistinct	Calcifications	More frequent than IDH-Mut	Foci of restricted diffusion
MGMT	Hemispheric; Meth → left side; UnMeth → right side	Meth indistinct	Meth → Low ITSS grade; UnMeth → High ITSS grade and more necrotic	Meth → Mixed-nodular; UnMeth → ring enhancement	Higher ADC values in Meth GBM
H3G34	Fronto-parietal lobe	Both defined and ill-defined	High T1w SI; calcification; heterogeneity	Subtle	Areas of ADC restriction

## Data Availability

Not applicable.

## References

[1] Louis D.N., Perry A., Reifenberger G., von Deimling A., Figarella-Branger D., Cavenee W.K., Ohgaki H., Wiestler O.D., Kleihues P., Ellison D.W. (2016). The 2016 World Health Organization Classification of Tumors of the Central Nervous System: A summary. Acta Neuropathol..

[2] Louis D.N., Perry A., Wesseling P., Brat D.J., Cree I.A., Figarella-Branger D., Hawkins C., Ng H.K., Pfister S.M., Reifenberger G. (2021). The 2021 WHO Classification of Tumors of the Central Nervous System: A summary. Neuro Oncol..

[3] Thompson C.B. (2009). Metabolic enzymes as oncogenes or tumor suppressors. N. Engl. J. Med..

[4] Camelo-Piragua S., Jansen M., Ganguly A., Kim J.C., Louis D.N., Nutt C.L. (2010). Mutant IDH1-specific immunohistochemistry distinguishes diffuse astrocytoma from astrocytosis. Acta Neuropathol..

[5] Reuss D.E., Mamatjan Y., Schrimpf D., Capper D., Hovestadt V., Kratz A., Sahm F., Koelsche C., Korshunov A., Olar A. (2015). IDH mutant diffuse and anaplastic astrocytomas have similar age at presentation and little difference in survival: A grading problem for WHO. Acta Neuropathol..

[6] Eckel-Passow J.E., Lachance D.H., Molinaro A.M., Walsh K.M., Decker P.A., Sicotte H., Pekmezci M., Rice T., Kosel M.L., Smirnov I.V. (2015). Glioma Groups Based on 1p/19q, IDH, and TERT Promoter Mutations in Tumors. N. Engl. J. Med..

[7] Horbinski C., Kofler J., Kelly L.M., Murdoch G.H., Nikiforova M.N. (2009). Diagnostic use of IDH1/2 mutation analysis in routine clinical testing of formalin-fixed, paraffin-embedded glioma tissues. J. Neuropathol. Exp. Neurol..

[8] Jenkins R.B., Blair H., Ballman K.V., Giannini C., Arusell R.M., Law M., Flynn H., Passe S., Felten S., Brown P.D. (2006). A t(1;19)(q10;p10) mediates the combined deletions of 1p and 19q and predicts a better prognosis of patients with oligodendroglioma. Cancer Res..

[9] Brat D.J., Aldape K., Colman H., Holland E.C., Louis D.N., Jenkins R.B., Kleinschmidt-DeMasters B.K., Perry A., Reifenberger G., Stupp R. (2018). cIMPACT-NOW update 3: Recommended diagnostic criteria for “Diffuse astrocytic glioma, IDHwildtype, with molecular features of glioblastoma, WHO grade IV”. Acta Neuropathol..

[10] Tesileanu C., Dirven L., Wijnenga M., Koekkoek J., Vincent A., Dubbink H.J., Atmodimedjo P.N., Kros J.M., van Duinen S.G., Smits M. (2019). Survival of diffuse astrocytic glioma, IDH1/2-wildtype, with molecular features of glioblastoma, WHO grade IV: A confirmation of the cIMPACT-NOW criteria. Neuro-Oncology.

[11] Yan H., Parsons D.W., Jin G., McLendon R., Rasheed B.A., Yuan W., Kos I., Batinic-Haberle I., Jones S., Riggins G.J. (2009). IDH1 and IDH2 mutations in gliomas. N. Engl. J. Med..

[12] Picart T., Barritault M., Poncet D., Berner L.P., Izquierdo C., Tabouret E., Figarella-Branger D., Idbaïh A., Bielle F., Bourg V. (2021). Characteristics of diffuse hemispheric gliomas, H3 G34-mutant in adults. Neuro-Oncol. Adv..

[13] Della Monica R., Cuomo M., Buonaiuto M., Costabile D., Franca R.A., del Basso de Caro M., Catapano G., Chiariotti L., Visconti R. (2022). MGMT and Whole-Genome DNA Methylation Impacts on Diagnosis, Prognosis and Therapy of Glioblastoma Multiforme. Int. J. Mol. Sci..

[14] Chen R., Smith-Cohn M., Cohen A.L., Colman H. (2017). Glioma Subclassifications and Their Clinical Significance. Neurotherapeutics.

[15] Habib A., Jovanovich N., Hoppe M., Ak M., Mamindla P.R., Colen R., Zinn P.O. (2021). MRI-Based Radiomics and Radiogenomics in the Management of Low-Grade Gliomas: Evaluating the Evidence for a Paradigm Shift. J. Clin. Med..

[16] Bhandari A., Sharma C., Ibrahim M., Riggs M., Jones R., Lasocki A. (2021). The role of 2-hydroxyglutarate magnetic resonance spectroscopy for the determination of isocitrate dehydrogenase status in lower grade gliomas versus glioblastoma: A systematic review and meta-analysis of diagnostic test accuracy. Neuroradiology.

[17] Lu J., Li X., Li H. (2021). Perfusion parameters derived from MRI for preoperative prediction of IDH mutation and MGMT promoter methylation status in glioblastomas. Magn. Reson. Imag..

[18] Thust S.C., Heiland S., Falini A., Jäger H.R., Waldman A.D., Sundgren P.C., Godi C., Katsaros V.K., Ramos A., Bargallo N. (2018). Glioma imaging in Europe: A survey of 220 centres and recommendations for best clinical practice. Eur. Radiol..

[19] Page M.J., McKenzie J.E., Bossuyt P.M., Boutron I., Hoffmann T.C., Mulrow C.D., Shamseer L., Tetzlaff J.M., Akl E.A., Brennan S.E. (2021). The PRISMA 2020 statement: An updated guideline for reporting systematic reviews. BMJ.

[20] Delfanti R.L., Piccioni D.E., Handwerker J., Bahrami N., Krishnan A., Karunamuni R., Hattangadi-Gluth J.A., Seibert T.M., Srikant A., Jones K.A. (2017). Imaging correlates for the 2016 update on WHO classification of grade II/III gliomas: Implications for IDH, 1p/19q and ATRX status. J. Neurooncol..

[21] Tang Q., Lian Y., Yu J., Wang Y., Shi Z., Chen L. (2017). Anatomic mapping of molecular subtypes in diffuse glioma. BMC Neurol..

[22] Darlix A., Deverdun J., Menjot de Champfleur N., Castan F., Zouaoui S., Rigau V., Fabbro M., Yordanova Y., Le Bars E., Bauchet L. (2017). IDH mutation and 1p19q codeletion distinguish two radiological patterns of diffuse low-grade gliomas. J. Neurooncol..

[23] Zhao K., Sun G., Wang Q., Xue Z., Liu G., Xia Y., Yao A., Zhao Y., You N., Yang C. (2020). The Diagnostic Value of Conventional MRI and CT Features in the Identification of the IDH1-Mutant and 1p/19q Co-Deletion in WHO Grade II Gliomas. Acad. Radiol..

[24] Park Y.W., Han K., Ahn S.S., Bae S., Choi Y.S., Chang J.H., Kim S.H., Kang S.G., Lee S.K. (2018). Prediction of *IDH1*-Mutation and 1p/19q-Codeletion Status Using Preoperative MR Imaging Phenotypes in Lower Grade Gliomas. AJNR Am. J. Neuroradiol..

[25] Wijnenga M., van der Voort S.R., French P.J., Klein S., Dubbink H.J., Dinjens W., Atmodimedjo P.N., de Groot M., Kros J.M., Schouten J.W. (2019). Differences in spatial distribution between WHO 2016 low-grade glioma molecular subgroups. Neuro-Oncol. Adv..

[26] Ellingson B.M., Cloughesy T.F., Pope W.B., Zaw T.M., Phillips H., Lalezari S., Nghiemphu P.L., Ibrahim H., Naeini K.M., Harris R.J. (2012). Anatomic localization of O6-methylguanine DNA methyltransferase (MGMT) promoter methylated and unmethylated tumors: A radiographic study in 358 de novo human glioblastomas. Neuroimage.

[27] Suh C.H., Kim H.S., Jung S.C., Choi C.G., Kim S.J. (2018). Clinically Relevant Imaging Features for MGMT Promoter Methylation in Multiple Glioblastoma Studies: A Systematic Review and Meta-Analysis. AJNR Am. J. Neuroradiol..

[28] Puntonet J., Dangouloff-Ros V., Saffroy R., Pagès M., Andreiuolo F., Grill J., Puget S., Boddaert N., Varlet P. (2018). Historadiological correlations in high-grade glioma with the histone 3.3 G34R mutation. J. Neuroradiol..

[29] Andreiuolo F., Lisner T., Zlocha J., Kramm C., Koch A., Bison B., Gareton A., Zanello M., Waha A., Varlet P. (2019). H3F3A-G34R mutant high grade neuroepithelial neoplasms with glial and dysplastic ganglion cell components. Acta Neuropathol. Commun..

[30] Yoshimoto K., Hatae R., Sangatsuda Y., Suzuki S.O., Hata N., Akagi Y., Kuga D., Hideki M., Yamashita K., Togao O. (2017). Prevalence and clinicopathological features of H3.3 G34-mutant high-grade gliomas: A retrospective study of 411 consecutive glioma cases in a single institution. Brain Tumor Pathol..

[31] Moon W.J., Choi J.W., Roh H.G., Lim S.D., Koh Y.C. (2012). Imaging parameters of high grade gliomas in relation to the MGMT promoter methylation status: The CT, diffusion tensor imaging, and perfusion MR imaging. Neuroradiology.

[32] Bernabéu-Sanz Á., Fuentes-Baile M., Alenda C. (2021). Main genetic differences in high-grade gliomas may present different MR imaging and MR spectroscopy correlates. Eur. Radiol..

[33] Kern M., Auer T.A., Picht T., Misch M., Wiener E. (2020). T2 mapping of molecular subtypes of WHO grade II/III gliomas. BMC Neurol..

[34] Du N., Zhou X., Mao R., Shu W., Xiao L., Ye Y., Xu X., Shen Y., Lin G., Fang X. (2022). Preoperative and Noninvasive Prediction of Gliomas Histopathological Grades and IDH Molecular Types Using Multiple MRI Characteristics. Front. Oncol..

[35] Lasocki A., Buckland M.E., Drummond K.J., Wei H., Xie J., Christie M., Neal A., Gaillard F. (2022). Conventional MRI features can predict the molecular subtype of adult grade 2–3 intracranial diffuse gliomas. Neuroradiology.

[36] Smits M. (2016). Imaging of oligodendroglioma. Br. J. Radiol..

[37] Foltyn M., Nieto Taborda K.N., Neuberger U., Brugnara G., Reinhardt A., Stichel D., Heiland S., Herold-Mende C., Unterberg A., Debus J. (2020). T2/FLAIR-mismatch sign for noninvasive detection of IDH-mutant 1p/19q non-codeleted gliomas: Validity and pathophysiology. Neurooncol. Adv..

[38] Corell A., Ferreyra Vega S., Hoefling N., Carstam L., Smits A., Olsson Bontell T., Björkman-Burtscher I.M., Carén H., Jakola A.S. (2020). The clinical significance of the T2-FLAIR mismatch sign in grade II and III gliomas: A population-based study. BMC Cancer.

[39] Patel S.H., Poisson L.M., Brat D.J., Zhou Y., Cooper L., Snuderl M., Thomas C., Franceschi A.M., Griffith B., Flanders A.E. (2017). T2-FLAIR Mismatch, an Imaging Biomarker for IDH and 1p/19q Status in Lower-grade Gliomas: A TCGA/TCIA Project. Clin. Cancer Res..

[40] Lee M.K., Park J.E., Jo Y., Park S.Y., Kim S.J., Kim H.S. (2020). Advanced imaging parameters improve the prediction of diffuse lower-grade gliomas subtype, IDH mutant with no 1p19q codeletion: Added value to the T2/FLAIR mismatch sign. Eur. Radiol..

[41] Kong L.W., Chen J., Zhao H., Yao K., Fang S.Y., Wang Z., Wang Y.Y., Li S.W. (2019). Intratumoral Susceptibility Signals Reflect Biomarker Status in Gliomas. Sci. Rep..

[42] Li W.B., Tang K., Zhang W., Yan W., You G., Li S.W., Zhang L., Huang Y.J., Jiang T. (2011). Relationship between magnetic resonance imaging and molecular pathology in patients with glioblastoma multiforme. Chin. Med. J..

[43] Yogananda C., Shah B.R., Nalawade S.S., Murugesan G.K., Yu F.F., Pinho M.C., Wagner B.C., Mickey B., Patel T.R., Fei B. (2021). MRI-Based Deep-Learning Method for Determining Glioma *MGMT* Promoter Methylation Status. AJNR Am. J. Neuroradiol..

[44] Ruiz-Ontañon P., Orgaz J.L., Aldaz B., Elosegui-Artola A., Martino J., Berciano M.T., Montero J.A., Grande L., Nogueira L., Diaz-Moralli S. (2013). Cellular plasticity confers migratory and invasive advantages to a population of glioblastoma-initiating cells that infiltrate peritumoral tissue. Stem Cells.

[45] Vettermann F.J., Felsberg J., Reifenberger G., Hasselblatt M., Forbrig R., Berding G., la Fougère C., Galldiks N., Schittenhelm J., Weis J. (2018). Characterization of Diffuse Gliomas with Histone H3-G34 Mutation by MRI and Dynamic 18F-FET PET. Clin. Nucl. Med..

[46] Hempel J.M., Brendle C., Bender B., Bier G., Skardelly M., Gepfner-Tuma I., Eckert F., Ernemann U., Schittenhelm J. (2018). Contrast enhancement predicting survival in integrated molecular subtypes of diffuse glioma: An observational cohort study. J. Neurooncol..

[47] Zhou H., Vallières M., Bai H.X., Su C., Tang H., Oldridge D., Zhang Z., Xiao B., Liao W., Tao Y. (2017). MRI features predict survival and molecular markers in diffuse lower-grade gliomas. Neuro Oncol..

[48] Castet F., Alanya E., Vidal N., Izquierdo C., Mesia C., Ducray F., Gil-Gil M., Bruna J. (2019). Contrast-enhancement in supratentorial low-grade gliomas: A classic prognostic factor in the molecular age. J. Neurooncol..

[49] Yamauchi T., Ohno M., Matsushita Y., Takahashi M., Miyakita Y., Kitagawa Y., Kondo E., Tsushita N., Satomi K., Yoshida A. (2018). Radiological characteristics based on isocitrate dehydrogenase mutations and 1p/19q codeletion in grade II and III gliomas. Brain Tumor Pathol..

[50] Feraco P., Bacci A., Ferrazza P., van den Hauwe L., Pertile R., Girlando S., Barbareschi M., Gagliardo C., Morganti A.G., Petralia B. (2020). Magnetic Resonance imaging Derived Biomarkers of IDH Mutation Status and Overall Survival in Grade III Astrocytomas. Diagnostics.

[51] Iliadis G., Kotoula V., Chatzisotiriou A., Televantou D., Eleftheraki A.G., Lambaki S., Misailidou D., Selviaridis P., Fountzilas G. (2012). Volumetric and MGMT parameters in glioblastoma patients: Survival analysis. BMC Cancer.

[52] Kanas V.G., Zacharaki E.I., Thomas G.A., Zinn P.O., Megalooikonomou V., Colen R.R. (2017). Learning MRI-based classification models for MGMT methylation status prediction in glioblastoma. Comput. Methods Programs Biomed..

[53] Korshunov A., Capper D., Reuss D., Schrimpf D., Ryzhova M., Hovestadt V., Sturm D., Meyer J., Jones C., Zheludkova O. (2016). Histologically distinct neuroepithelial tumors with histone 3 G34 mutation are molecularly similar and comprise a single nosologic entity. Acta Neuropathol..

[54] Thust S.C., Hassanein S., Bisdas S., Rees J.H., Hyare H., Maynard J.A., Brandner S., Tur C., Jäger H.R., Yousry T.A. (2018). Apparent diffusion coefficient for molecular subtyping of non-gadolinium-enhancing WHO grade II/III glioma: Volumetric segmentation versus two-dimensional region of interest analysis. Eur. Radiol..

[55] Pruis I.J., Koene S.R., van der Voort S.R., Incekara F., Vincent A., van den Bent M.J., Lycklama À., Nijeholt G.J., Nandoe Tewarie R., Veldhuijzen van Zanten S. (2022). Noninvasive differentiation of molecular subtypes of adult nonenhancing glioma using MRI perfusion and diffusion parameters. Neuro-Oncol. Adv..

[56] Xing Z., Huang W., Su Y., Yang X., Zhou X., Cao D. (2022). Non-invasive prediction of p53 and Ki-67 labelling indices and O-6-methylguanine-DNA methyltransferase promoter methylation status in adult patients with isocitrate dehydrogenase wild-type glioblastomas using diffusion-weighted imaging and dynamic susceptibility contrast-enhanced perfusion-weighted imaging combined with conventional MRI. Clin. Radiol..

[57] Han Y., Yan L.F., Wang X.B., Sun Y.Z., Zhang X., Liu Z.C., Nan H.Y., Hu Y.C., Yang Y., Zhang J. (2018). Structural and advanced imaging in predicting MGMT promoter methylation of primary glioblastoma: A region of interest based analysis. BMC Cancer.

